# Emerging Methods in Biosensing of Immunoglobin G—A Review

**DOI:** 10.3390/s23020676

**Published:** 2023-01-06

**Authors:** Tehmina Azam, Syed Hassan Bukhari, Usman Liaqat, Waheed Miran

**Affiliations:** 1 School of Chemical and Materials Engineering (SCME), National University of Sciences and Technology (NUST), Islamabad 44000, Pakistan; 2 College of Computational Sciences and Natural Sciences, Minerva University, San Francisco, CA 94103, USA

**Keywords:** immunoglobin G (IgG), biosensors, IgG detection, limit of detection (LOD), electrochemical sensors

## Abstract

Human antibodies are produced due to the activation of immune system components upon exposure to an external agent or antigen. Human antibody G, or immunoglobin G (IgG), accounts for 75% of total serum antibody content. IgG controls several infections by eradicating disease-causing pathogens from the body through complementary interactions with toxins. Additionally, IgG is an important diagnostic tool for certain pathological conditions, such as autoimmune hepatitis, hepatitis B virus (HBV), chickenpox and MMR (measles, mumps, and rubella), and coronavirus-induced disease 19 (COVID-19). As an important biomarker, IgG has sparked interest in conducting research to produce robust, sensitive, selective, and economical biosensors for its detection. To date, researchers have used different strategies and explored various materials from macro- to nanoscale to be used in IgG biosensing. In this review, emerging biosensors for IgG detection have been reviewed along with their detection limits, especially electrochemical biosensors that, when coupled with nanomaterials, can help to achieve the characteristics of a reliable IgG biosensor. Furthermore, this review can assist scientists in developing strategies for future research not only for IgG biosensors but also for the development of other biosensing systems for diverse targets.

## 1. Introduction

### 1.1. Introduction to Immunoglobins and Immunoglobin G (IgG)

**Figure 1:**
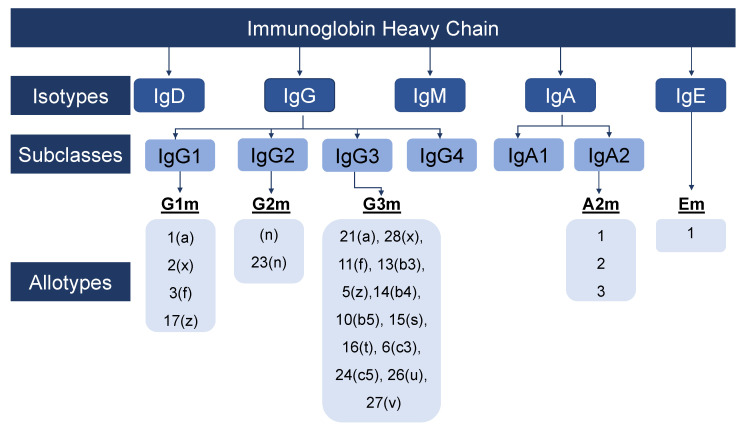
The diagram shows the isotypes, subclasses, and allotypes of immunoglobulins in humans with respective letters and numbers assigned to each type and subtypes of immunoglobulins. Adopted and used with permission from [9].

Antibody or immunoglobin (Ig) is a small protective protein produced by the human immune system upon the manifestation of foreign substances known as antigens and constitutes 20% of plasma protein. Immunoglobins eradicate new antigens in the body by recognizing and binding with them. There are multiple kinds of antigens: They could be viruses, bacteria, toxins, etc., and these antibodies instruct plasma cells to produce specific antibodies [1]. Antibodies were discovered due to the work of Emil von Behring’s discovery of antitoxins for diphtheria, tetanus, and anthrax [2]. Antibodies are further classified into five diverse types: Immunoglobin M (IgM), Immunoglobin D (IgD), Immunoglobin G (IgG), Immunoglobin A (IgA), and Immunoglobin E (IgE), as shown in Figure 1. These couple glycoproteins are composed of 82–96% protein and 4–18% carbohydrate and differ in heavy chain structure with varying effector functions [3].

IgG alone accounts for about 10–20% of plasma proteins and 70% to 75% of the total immunoglobulins in plasma, making it one of the primary antibodies and highest concentrations of proteins in human serum [4]. The subclasses of IgG can further be categorized on the basis of decreasing abundance of Immunoglobin G 1 (IgG1), Immunoglobin G 2 (IgG2), Immunoglobin G 3 (IgG3), and Immunoglobin G 4 (IgG4), which differ based on varying primary amino acid sequences and structural differences in the hinge and heavy chain constant regions [5,6,7]. The basic structure of an antibody contains two light chains (LCs) which are similar and two indistinguishable heavy chains (HCs) while making a Y-shaped compact structure composed of two heterodimers (linked together via a disulfide bridge). Human HCs for each subtype of an antibody differ from each other, and LCs have a constant region (CL) plus a variable region (VL) [8].

#### 1.1.1. The Basic Biology of IgG

The molecular mass of IgG is approximately 160 kDa, which happens to be due to the presence of light and heavy chains each being two in number [10,11]. The fragment antigen binding (Fab) region present inside the IgG structure contains a paratope and can help in pathogen inhibition through recognition events whereas the fragment crystallizable (Fc) region is in charge of the interaction with different accessory molecules to moderate indirect effector operations, including the complement–dependent cytotoxicity mechanism (CDC), antibody-dependent cellular cytotoxicity, and phagocytosis (ADCC and ADCP) [12,13]. 

**Figure 2:**
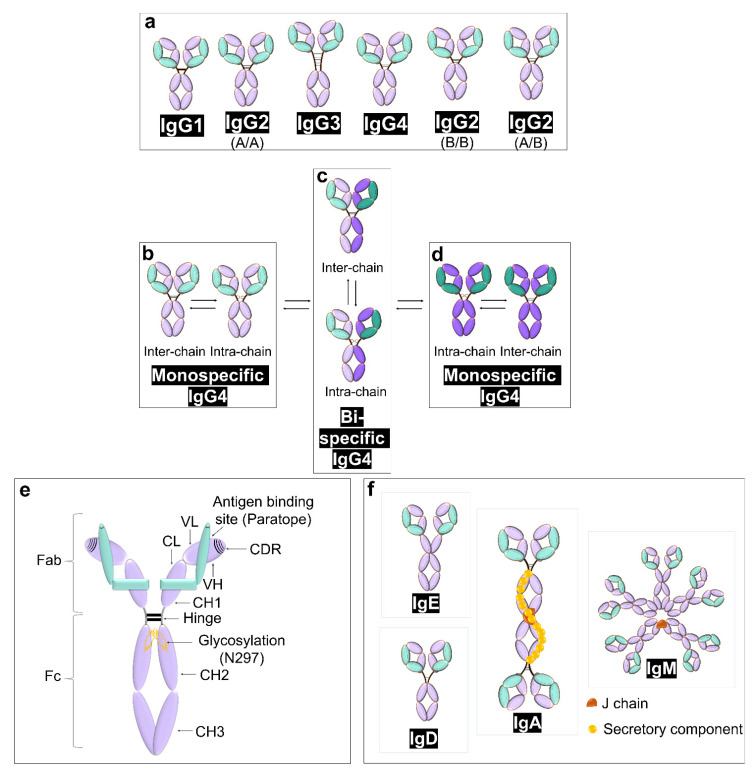
Differences in the structures of IgG subtypes and allotypes (**a**–**d**), adapted with permission from [14]. Some of the differences are due to hinge region while others are unique due to their heavy and light chains. Detailed insight of the regions of IgG. (**e**) The light chain (shown in green) vs. the heavy chain (shown in purple). Antigen specificity is determined by complementarity determining regions (CDR, striped lines). The glycosylation patterns are shown in yellow, as they also have impact on the function of IgG, adapted with permission from [9]. The comparison of IgG structure can be made with other immunoglobins shown in (**f**), adapted with permission from [15].

Extensive research in the 20th century led to the discovery of IgG subclasses in the 1960s by using rabbit antisera specific to human IgG myeloma proteins [3,7]. Although there is a 90% similarity between IgG subclasses at an amino acid level, they differ with respect to their antigen binding event, half-life, formation of immune complex, placental transport, and effector cell triggering [14]. The detailed differences in the structure of IgG subtypes and allotypes have been shown in Figure 2a–d along with regions and various components of IgG (Figure 2e), and schematics for the structure of other immunoglobins (IgE, IgD, IgA, and IgM) have also been shown (Figure 2f).

#### 1.1.2. Importance of IgG

The modern world has improved the resolution, throughput, and sensitivity of the instrumentation needed for analyzing glycoproteins. However, even with these advancements, precision in instrumentation still suffers [16]. IgG affects therapeutic antibodies, and the same effect is passed on to our immune systems, which play a part in protection against pathogens [16,17]. Serological tests are simpler to develop, more practical, and provide faster results [18]. Advancements in serological tests have marked immunoglobins, e.g., IgM, IgG, and IgA, etc., as important biomarker tools [19,20]. Moreover, our literature review identifies positive results with IgG and IgM tests either exclusively or combined [21]. Recent studies emphasized the importance of glycosylation in the therapeutic effects of IVIg [22,23]. Further evidence identifying anti-inflammatory properties has been shown in mouse models [24,25]. The importance of IgG is further characterized by improving immunity, treatment of pathological diseases and biomarkers, and its role in vaccines and biosensors for diagnostics.

#### 1.1.3. Role of IgG in Immunity

**Figure 3:**
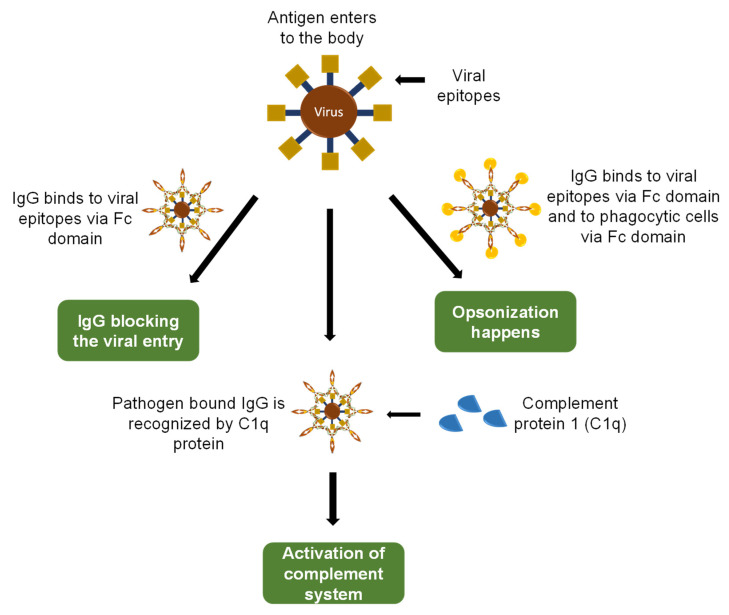
The first mechanism is the neutralization of the pathogen’s ability to enter host cells, and toxins are stopped to enter the host cells. IgG binds itself to viral epitopes via Fab domain. This event is responsible for the blockage of vial entry, fusion, and maturation inside the host cells. The second mechanism is the binding of antigen-bound IgG with phagocytic cells, which leads to the ingestion of both viral particles and phagocytes; this mechanism is known as opsonization. The last mechanism involves the recognition of antigen-bound IgG molecules by complement proteins (Cq1) and activation of complement system, which leads to virus eradication.

Based on the kind of molecule involved in providing the defense mechanism against foreign entities, human immunity has been categorized into two types, i.e., cell-mediated immunity and humoral immunity, where cell-mediated immunity involves the role of T cells while humoral immunity works because of antibodies [26,27]. IgG is an important class of antibodies that comprises 75% of all the immunoglobins present in the plasma and plays its function in humoral immunity [28]. The ability of IgG to bind with a diverse number of molecules comes from the primary amino acid sequences, hence diversifying the capability of IgG to tackle pathogens. The general defense mechanism of IgG against pathogens is a different mechanism, and three of them are depicted in Figure 3. 

#### 1.1.4. IgG as an Important Biomarker and Different Pathological Conditions 

As evident from the literature, IgG is an important biomarker for multiple pathological conditions. The recent onset of SARS-CoV-2 once again shifted the focus onto IgG serum level studies, as it fluctuates upon the infection. Baoqing et al., have studied responses of IgG and IgM glycoproteins in COVID-19 patients and elevations in levels of N and S protein (of virus)-specific IgG and IgM after the onset of symptoms in non-ICU (nonintensive care unit) patients [29]. Because serological methods cannot be used in preliminary stages of the infection (up to the sixth day), they can make the diagnosis in the later stages (from the ninth day to the third month) of the disease. Therefore, IgG can be used to understand that people who do not show symptoms have had this disease rather than a COVID-19 diagnosis. Zhang et al., reported that the isotyping of immune response based on IgG response and NLR (neutrophil to lymphocyte ratio) can be a useful tool to differentiate COVID-19 patients on the foundation of disease severity [30]. Hongyan et al., stated that the quantification of IgG antibodies can be an important tool to evaluate the prognosis and seriousness of COVID-19 infection [31]. 

Apart from that, MOG-IgG has been confirmed to have a link with neuromyelitis optica (NMO), a central nervous system disorder [14]. Stefan et al., studied the correlation between serum IgG levels and efficiency in checkpoint inhibition and reported that IgG and its subclasses could serve as an important biomarker in metastatic melanoma patients to predict the success of checkpoint inhibition [2]. Dani et al., have reported the association of IgG serum levels with Shigella (a type of bacteria that is the leading cause of diarrhea and death related to this disease), and identification of the threshold level of these protective antibodies can help in the development of vaccines for children as well as infants. During infection, IgG is produced to enhance serotype-specific immunity, and levels of antibodies fluctuated in dose-response manners when different vaccines were tested under phase three studies and CHIM (controlled human infection model) clinical trials [32]. In addition to that, the presence of serum antibodies has also been reported as an important hallmark for neuromyelitis optica (NMO) [33]. So, it can be inferred that high or low levels of serum IgG are associated with a broad spectrum of diseases and can be used as an important biomarker that emphasizes the importance of IgG biosensing. 

### 1.2. Introduction to Biosensors

**Figure 4:**
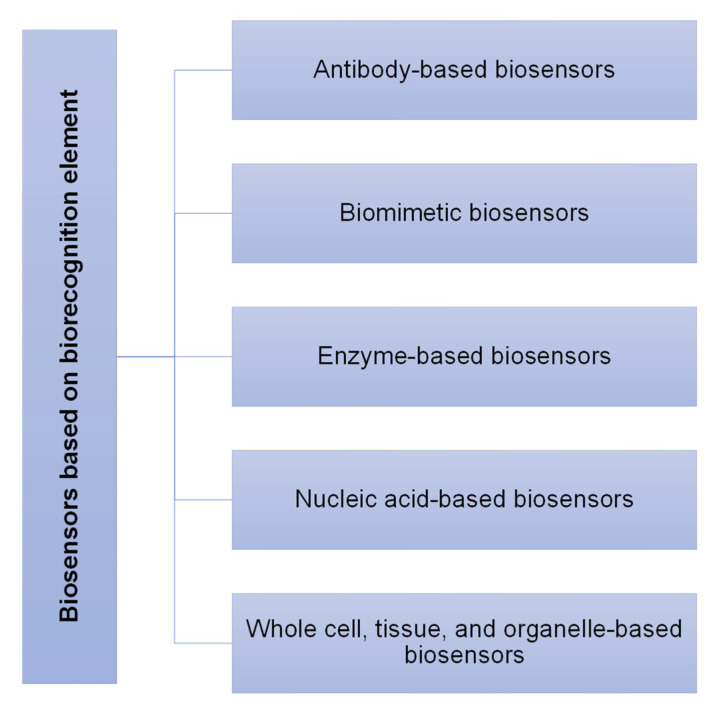
Classification of biosensors based on biorecognition elements.

**Figure 5:**
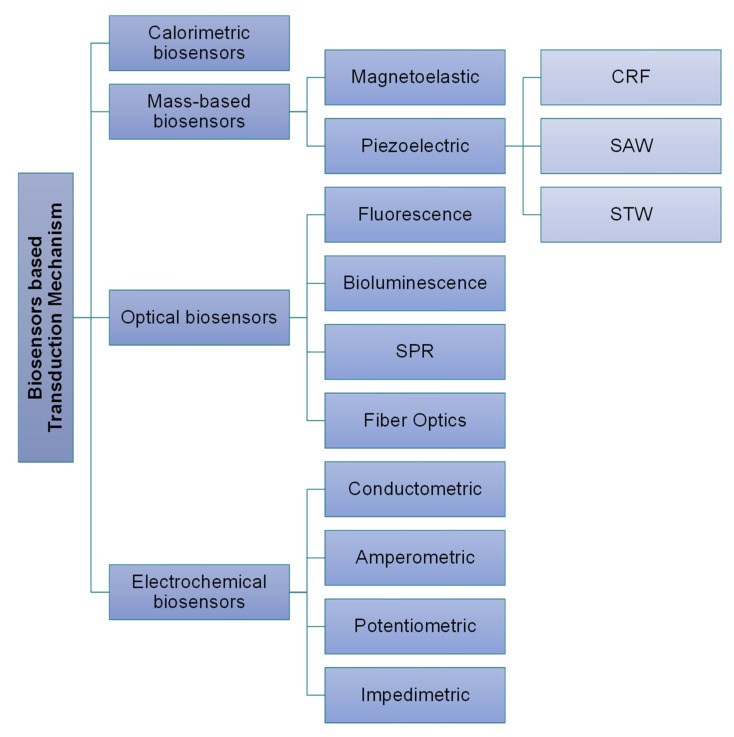
Classification of biosensors based on transduction mechanisms.

**Figure 6:**
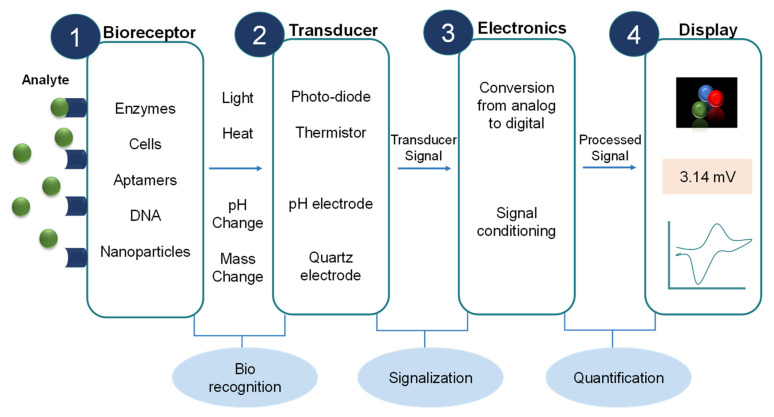
The components and working principle of a typical biosensor represent levels of components, the types of components, and the purpose of each level. For instance, a bioreceptor is a component with types, such as aptamers, enzymes, etc., with the purpose of biorecognition. Adapted from [37].

Biosensors are generally referred to as small but sensitive devices that measure biomolecules (also known as an analyte) with the help of multiple output signals generated by various chemical or biological reactions [34]. The usage of biosensors has been in the healthcare sector, food industry, and environmental monitoring for a few decades back [35]. In the healthcare industry, biosensors are being used during both the diagnosis and treatment stages of any disease as well as in research labs [36]. The working principle of a biosensor is highlighted by the main components of a typical biosensor, and these components are as follows [37]:

- Analyte or substrate: A biomolecule that needs to be recognized, i.e., IgG antibody, is an analyte that can be detected via various biosensors.
- Receptor or biorecognition elements: These are the molecules that have an affinity to bind to the analyte. Enzymes, antibodies, antiantibodies, aptamers (DNA or RNA), and proteins are a few examples of receptors. The phenomenon of the affinity binding of an analyte to a receptor is known as biorecognition. As biorecognition elements are specific to their analyte and according to the need of the time, one more biorecognition element is used in a biosensor, and based on this, biosensors can be divided into different categories as listed in Figure 4 [38].

- Transducer: Transducers help in the conversion of the biorecognition phenomenon into a measurable output signal, and this process is known as the transduction mechanism or signalization. Based on multiple transduction events happening in various biosensors, they have been classified as shown in Figure 5 [39].
- Electronics: Electronics are the part of a biosensor that deals with the processing of the transduction mechanism and prepares it for display. Complex electronic circuits work here that help in the amplification of transduction signals from analog to digital form.
- Display: Presenting the data in a readable form is referred to as the display, which is a combination of both hardware and software, and it displays output signals in accordance with the user’s need either in the form of graphs, numeric, images, or tables, and complete schematics of a biosensor component are shown in Figure 6.

## 2. Biosensors for the Detection of IgG

The effort to develop an IgG biosensor has been there for a few decades, and it was further highlighted due to the COVID-19 outbreak in 2019 because of the strong link between serum IgG titer and SARS-CoV-2 infection. Some of the biosensors are highlighted in Table 1. 

### 2.1. Electrochemical Biosensors for IgG Detection

**Figure 7:**
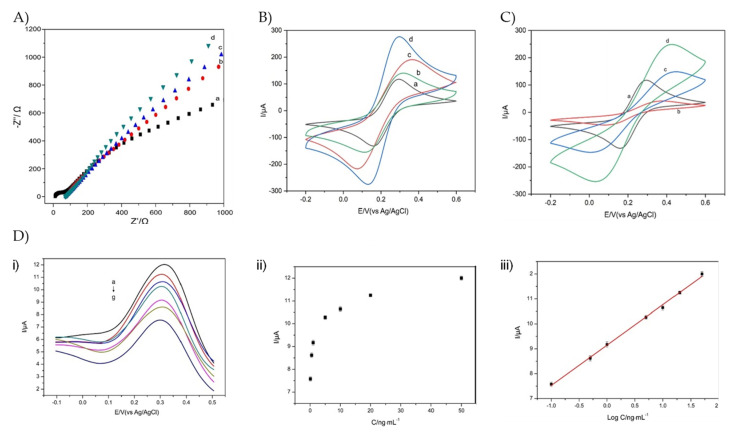
In figure (**A**–**C**), the samples were bare GCE (a), MoS2/GCE (b), MoS2@N− GQDs/GCE (c), and MoS2@N−GQDs−IL/GCE (d), giving different Nyquist plots in (**A**) and voltammograms in (**B**) and (**C**). In (**D**) (**i**) shows the differential pulse voltammograms with varying concentrations of MIPs NPs/MoS2@N−GQDs−IL/GCE electrode in PBS while in (**D**) (**ii**,**iii**) shows the calibration curves for human IgG at MIPs NPs/MoS2@N−GQDs−IL/GCE. Results were used with permission from [57].

Electrochemical biosensing has an enormous impact on the biosensing of IgG, and a broad range of studies have been conducted to attain the unique, selective, sensitive, and economical characteristics of a biosensor. Liang et al., has reported a molecular imprinted electrochemical biosensor for selective and extremely sensitive detection of IgG [57]. The GCE (glassy carbon electrode) was modified using a MoS2@N-GQDs-IL (molybdenum disulfide @nitrogen-doped graphene quantum dots with ionic liquid) nanocomposite. Further testing was carried out by using cyclic voltammetry (CV), EIS (electrochemical impedance spectroscopy), and DPV (differential pulse voltammetry), and some of the results are compiled in Figure 7 [57]. 

The recent outbreak of COVID-19 had impacted scientists in a way to explore economic, rapid, sensitive, and selective methods for the detection of viral particles. During that point in time and up until now, nanomaterials have been explored to achieve qualities in a biosensor. Meanwhile, the direct detection of IgG antibodies to SARS-CoV-2 in blood using an incredibly accurate label-free nanosensor based on integrated graphene and Au nanostars was proposed by Seyyed et al. This platform was unique and gives a response for IgG produced against SARS-CoV-2 spike protein within a minute [58]. Likewise, PDA-AgNPs-PDA-Au (polydopamine silver nanoparticles polydopamine gold) film has been developed for the detection of horse IgG [59]. Other than that, a number of other electrochemical sensors have been proposed until today for the detection of IgG, as listed in the sections given below. 

#### 2.1.1. Potentiostatic Biosensors for IgG Detection

Potentiostatic biosensors are the type of electrochemical biosensor in which the potentiostat detects the voltage difference between the working electrode (WE) and reference electrode (RE) by adjusting the potential difference of the counter electrode with respect to the WE while the resultant potential difference is determined by the concentration of an analyte [60,61,62]. These sensors are an important type known as redox-potential biosensors because the redox reaction is the main principle governing these sensors. The response towards an analyte is determined by ion conduction and ion exchange reactions at the solution interface/membrane; hence, the measurement of the free ions via these biosensors requires calibration on regular basis [63]. A light addressable potentiometric sensor (LAPS) was proposed by Jintao et al., based on the covalently functionalized membrane for the detection of IgG. A linear relationship between the potential shift and the concentration of IgG was observed, hence reporting a detection range of 0–150 μg/mL [64]. 

#### 2.1.2. Conductometric Biosensors for IgG Sensing 

Conductometric sensors measure the conductivity difference occurring due to a biological event in an analyte solution [65]. It can detect both electroactive and electroactive species, and the conductivity electrodes can be in direct contact with the solution or insulated by a thin layer [66]. Conductometric sensors have been widely used, particularly as gas sensors and for evaluating engine oil quality. However, their use in microbial detection in food has been restricted [67]. Conductometric biosensors are useful because they work at a low working voltage, do not require the use of a reference electrode, perform quick analysis, have a reasonably high sensitivity and precision for detection, and can be manufactured using low-cost technology [68,69,70]. 

The conducting polymer, which works as an electrochemical transducer to transform biological impulses into electrical signals, is an essential component of a conductometric biosensor. A few examples of conducting polymers are polyaniline, polythiophene, polyacetylene, and polypyrrole (PPy) [71,72]. As explained in the following literature review, these conducting polymers have distinct and extensive uses in the medical field. Polyaniline may be used to detect cholesterol in blood via an enzymatic approach, and nonenzymatic detection of IgG in humans is viable by utilizing polyaniline gold nanospheres as nanolabels [73,74]. Noninvasive usage of a PPy layer on the gold surfaces of disposable screen-printed electrodes (SPE) utilizing 1H-pyrrole-1-propanoic acid revealed new applications for conductometric biosensors [75]. Despite the fact that this type of biosensing is noninvasive and research is being conducted on nonenzymatic forms of detection, such as cholesterol detection, the current market uses an enzymatic form of detection, which has some drawbacks in the form of fluctuations in conductivity due to enzymatic reactions and the conductivity of the electrolyte solution [76]. Okafor et al., proposed a conductometric biosensor based on polyaniline (PANI) for the detection of Johne’s disease-specified IgG antibodies [77]. This sensor was further compared with the detection of IgG with ELISA, and this study confirmed the moderate agreement between both techniques [78]. A single PANI wire was used to propose yet another conductometric biosensor for the detection of IgG and myoglobin with an LOD of 3ng/mL for IgG [79]. 

Nonetheless, the conductometric technique gives remarkable results in IgG detection, particularly when the technology is repurposed from contaminants in the environment to pollutants in the human body in the future.

#### 2.1.3. Impedimetric Biosensors for IgG Detection 

Impedimetric biosensors are electrochemical sensors that measure changes in capacitance and conductance on the surface of an electrode [80]. Based on the type of biorecognition element, impedimetric biosensors can be categorized into cell-based impedimetric biosensors, immunobinding-based, nucleic acid-based, and enzyme-based biosensors [81]. They have been used to measure multiple biological molecules because of the unique properties of each type of impedimetric biosensor, i.e., sensitivity, selectivity, and reproducibility [82]. As mentioned above the output signal for this type of biosensor is the impedance of conductance, and these signals are always proportional to the concentration and activity of the analyte [83] as studied by Zou et al. They have explored the role of a nanointerdigitated electrode array (nIDA) coupled with a microfluidic system integrated onto a polymer substrate which can be used for impedimetric biosensing of protein, cells, and genes [84]. 

**Figure 8:**
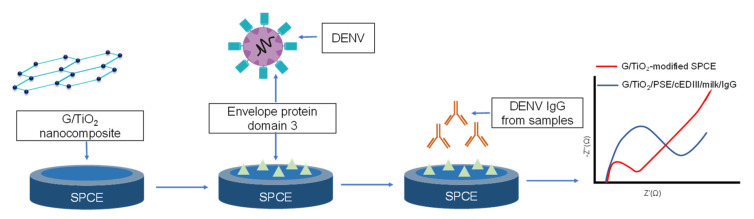
An impedimetric sensor for detecting DENV IgG proteins using G/TiO₂/PSE-modified nanocomposite. The graph represents a Nyquist plot with the red indicating G/TiO₂-modified SPCE and the blue indicating G/TiO_2_/PSE/cEDIII/milk/IgG. Used with permission from [86].

During the early 2000s, it was reported that impedimetric biosensors are less frequent. Still, studies are reported to use these sensors for sensitive sensing, as the hybridization of DNA was monitored via this category of sensors. In addition, a very low concentration of about 10 pg/mL of antibodies was detected via an impedimetric biosensor that used polypyrrol film [85]. Furthermore, multiple studies have reported impedimetric sensors for IgG detection specifically. In this regard, a dengue virus IgG impedimetric biosensor based on a screen-printed glassy carbon electrode has shown satisfactory performance up to a minimum concentration of 2.81 ng/mL of viral IgG antibodies. The general schematics of this sensor have been shown in Figure 8 along with the graph of signals generated for the biosensor [86]. 

Similarly, Schrattenecker et al., have introduced the generation of a redox pair Hexaamineruthenium (II)(III) during impedimetric studies via a gold electrode, and the detection range of IgG for this specific sensor was 11.3 ng/mL to 113 μg/mL. The use of an alternative redox couple for an impedimetric biosensor with high stability was proposed for a stable IgG biosensor with redox-probe Hexaammineruthenium (II)/(III) [87]. Protein G has a binding affinity with IgG, so this interaction of both proteins was used to develop a highly sensitive biosensor with a limit of detection up to 1 × 10^–14^ mol/L [41]. Moreover, measles-specific primary IgG antibodies have been reported to be sensed via an impedimetric immunosensor with the help of a label-free approach [42]. Another label-free impedimetric biosensor was proposed by Honglan et al., with an LOD of 5 ng·mL^−1^ and a linear range of 10 ng·mL^−1^ to 1.0 μg·mL^−1^ [88]. Bhasin et at. proposed a simple dipping and reading model for the detection of proteins. “O^2^VBR” increases signal amplitudes by irreversibly oxidizing the VBR (virus bioresistor) channel for the detection of DJ-1 and two different IgG antibodies. IgG antiglycan antibody detection was made possible using impedimetric biosensors on a GPI phosphoglycan bioreceptor with an LOD of 0.31 IU mL^−1^ [89]. 

#### 2.1.4. Amperometric Biosensors for IgG Detection

Similar to other types of electrochemical biosensors, amperometric biosensors generate the signals after an electrochemical event, but their output signals are in the form of either current or potential. The change in current or voltage fluctuates based on the analyte concentration, as studied by Malhotra et al.; the amperometric biosensor’s working electrode is typically made of either a noble metal (gold, titanium, nickel, etc.), ITO (indium tin oxide), or coating of carbon with bioreceptor elements. Upon applying the potential, the current is produced via enzymatic conversion or absorption of proteins at the surface of an electrode [90]. 

The approach of amperometric sensing for IgG is an old one, and most work has been performed since the 2000s. Dutra et al., described the importance of epoxy graphite biocomposite consisting of silver and TCNQ (tetracyanoquidimethane). This material has enhanced the selectivity of IgG sensing by reducing the potential down to 0.28 V [91]. 

The detection of rabbit IgG as a model analyte has been developed utilizing a separation-free homogenous immunosensor that is disposable and mediator-free and is based on a conducting polymer-covered, screen-printed carbon electrode. This sensor’s foundation was an analysis of the accessible antibody binding sites using free and tagged antigens in a competitive experiment. The catalytic current was measured amperometrically at/0.35 V vs. Ag/AgCl and revealed a linear range of RIgG concentrations ranging from 0.5 to 2 mg/mL with a standard deviation of 9/0.0145. The detection threshold was found to be 0.33 mg/mL [92]. Based on improved stiff biocomposites, highly sensitive electrochemical amperometric immunosensors for IgG detection have also been reported [44]. IgG regulation is not only important for the human body but also plays a significant role in monitoring FPT (failure of passive transfer) in calves. Hence, Robinson et al., discussed the creation of a label-free impedimetric immunosensor for bovine IgG in serum and showed how it might be used to estimate the FPT in newborn calves. The designed sensors successfully distinguished between newborn calf sera both pre- and postcolostrum feeding and showed quick and accurate IgG detection. Such technology might make it possible to determine FPT quickly, enhancing the vigor and survival rate of calves [93]. 

### 2.2. Mass-Based Biosensors

#### 2.2.1. Magnetoelectric Biosensors 

Magnetoelectric biosensors work on the principle of the magnetoelectric effect which refers to the fluctuation in a material’s electric polarization due to the applied magnetic field (direct effect) or change in the magnetic field under the influence of an electric field (converse effect). The magnetoelectric effect is prominent in materials that exhibit ferromagnetic and ferroelectric properties simultaneously, known as multiferroic materials. The following effect is also prominent if a material acquires electric polarization under the action of a magnetic field [94]. 

The ME effect was first observed experimentally in the single-phase multiferroic material Cr_2_O_3_ in 1961. Numerous studies were then conducted all over the world to improve the coupling capability of ferroelectric and magnetic orderings in a single-phase material system [95]. Combining magnetoelectric detection methods with other techniques helps in the extremely sensitive detection of biomolecules. As reported by Mulvaney et al., combined chip-based magnetoelectric biosensing with FFD (fluidic force discrimination) gave high sensitivity to the detection of biomolecules, including IgG, in plasma up to femtomolar concentrations with an incubation time of only 5 min for a single protein [96].

#### 2.2.2. Piezoelectric Biosensors

Piezoelectricity, also known as the piezoelectric effect, is a physical phenomenon that describes a material’s ability to produce voltage when mechanically stressed. The effect is also effective in the opposite situation. When a voltage is applied to the surface of a piezoelectric material, it causes mechanical stress or oscillation. Anisotropic crystals, or crystals with no center of symmetry, are typical piezoelectric materials [97]. In comparison to methods such as surface plasmon resonance, piezoelectric biosensors have low manufacturing costs and simple assay mechanisms. These biosensors might attract users because of their ability to detect macromolecules in diagnosis, and further improvements can increase or enhance their application [98]. A piezoelectric platform was developed for the attachment of IgGNS1 to detect the nonstructural protein of dengue fever. This system easily detected NS1 protein levels only up to 10-fold dilutions [99,100]. 

#### 2.2.3. Quartz Crystal Microbalance (QCM)

The quartz crystal microbalance (QCM) refers to a biosensing platform with a mechanical transduction part and operates under the rule of mass detection. Recent years have been benchmark years for this platform because of its unique ability to virtually detect any biomolecule in a label-free mode [101]. These biosensors are rapid and sensitive for diagnostic purposes [102]. 

Using an external electric field to apply to quartz results in mechanical stresses in the crystal according to the piezoelectric effect, which is the basis for QCM. The crystal oscillates in a direction perpendicular to the plate surface when an alternating voltage is applied to it [102,103,104]. Numerous inherent advantages exist for disease biosensing with the QCM mass-based detection principle. Since mass is a fundamental property of all matter, QCM systems can detect almost any kind of molecule, making them an ideal platform for identifying the various kinds of disease biomarkers. The frequency response instantly reflects molecular recognition events at the crystal surface without the need for labeling procedures, leading to a quick detection time that is typically between 30 min and 1 h [102]. 

According to the literature, QCM has been used to improve the binding of protein A and IgG molecules, SAM molecules and protein A, and other compositions for better disease biosensing, e.g., through the coating of the gold surface of the quartz crystal with a 35 nm polystyrene film and then treating it with an acid [105,106]. Similar to the combined use of aptamers and antibodies mentioned in the Biosensors Based on Biorecognition Element section, QCM can utilize enzyme-based methodology coupled with antibodies for improving the sensitivity of the amplification system by using anti-IgG–horseradish peroxidase enzyme conjugate [107]. In this case, although the detection of IgG is limited, it opens doors to show IgG detection could be the next step with simple modifications and appropriate coupling of the biorecognition element, hence proving that QCM has the potential for IgG detection. Kata et al., designed, fabricated, and characterized QCM biosensors based on a multichannel monolithic platform. Anti-IgG antibodies were immobilized to target IgG detection. This platform was reported to be label-free with real-time detection [108]. In another study, QCM was coupled with protein A, and the determined LOD was 5.07 pg/mL (standard deviation of 0.18pg/mL) with a linear range of 5.0 pg/mL–20.0 ng/mL [109]. Dai et al., reported that, with the addition of hexacyanoferrate and iron(III) ions at pH 4, the growth of “snow ball”-like objects greatly enhanced the response of a QCM biosensor, providing an LOD of 2.4 nM, which was facilitated by a surface-captured analyte (IgG from urine) [110]. 

#### 2.2.4. Surface Acoustic Waves (SAW)

The manipulation of surface acoustic waves results in yet another type of mass-based biosensing (SAWs). SAWs are elastic waves that spread across the surface of a solid using piezoelectric crystals. They are similar to ocean waves in that they are guided by an electromechanical coupling, and SAW devices are widely used in communication and sensing [111,112]. They can record remarkably small frequency shifts brought on by incredibly small mass loadings because their frequency ranges from several hundred MHz to GHz [113]. 

SAWs have been utilized in biosensors due to their ability to miniaturize designs with high thermal stability, high sensitivity, quick response times, low cost, ease of fabrication, and wire-free integration [113,114,115]. Due to their special qualities, they are widely regarded as smart transducers that can be combined with a wide range of recognition layers, including functional polymers, carbon nanotubes, graphene sheets, metal oxide coatings, and biological receptors [115,116,117]. SAW sensors can therefore detect a wide variety of sensing targets, including small gas molecules, large bioanalytes, and even entire cell structures [113].

With developments in biosensing technology, SAW has been developed with precise control for promising utility in biosensors. For instance, the shear-horizontal surface acoustic wave (SH-SAW) biosensor is an inventive pathogen detection platform for detecting the anti-SARS-CoV-2 nucleocapsid antibody (IgG-based) [118]. Although there are still cases of poor sensitivity and an inability to detect small molecules, Yao et al., developed a method for quantifying Escherichia coli L-asparaginase by immobilizing amide-coupled polyclonal antibodies, which significantly improved sensitivity [119,120]. Although compared to other biosensing techniques, SAWs have certain drawbacks, as they stand to be a relatively modern method of biosensing; nonetheless, they stand their ground with new advancements [111].

### 2.3. Biosensors Based on Biorecognition Element

Biosensors are also categorized based on the biorecognition element used to modify the transducer of biosensors. Some of these sensors are compiled in Table 2 while the rest of the explanation has been provided in upcoming sections of the paper. 

#### 2.3.1. Antibodies-Based IgG Detection

An innovative type of antiviral intervention is through monoclonal antibodies (mAbs) that can attach to and “neutralize” a virus in infected people [131]. Neutralizing mAbs are recombinant proteins generated from convalescent patients or humanized-mice B cells. High-throughput screening of these B cells enables the discovery of antibodies with the specificity and affinity to attach to a virus and inhibit viral entrance, hence eliminating disease associated with productive infection. These mAbs are referred to as “neutralizing”. They can eventually be utilized as passive immunotherapy (described later) to reduce virulence [131,132]. 

These antibodies are versatile and are employed as a biorecognition material in biosensors to create antibody-based biosensors or immunosensors, which employ a transducer to convert the antibody–antigen binding event to a quantifiable physical signal [133]. Immunosensors have transformed diagnostics by detecting a variety of analytes, including disease indicators, dietary and environmental toxins, biological warfare agents, and illegal substances [134]. Immunosensors are classified into two types: nonlabelled immunosensors and labeled immunosensors. Non-labeled immunosensors are constructed so that the antigen–antibody complex may be identified immediately by detecting the physical changes caused by the complex’s creation. A tagged immunosensor, on the other hand, incorporates a sensitively detectable label. The immunocomplex is thus determined sensitively using label measurement [135]. Immunosensors are frequently employed in clinical diagnostics to detect or quantify disease-related chemicals, such as carcinogenic molecules, proteins, LDLs, food pathogens, bacteria, and viruses, because of their higher affinity of the antibody–antigen complex with high selectivity [90,136]. Recent advances in immunosensors have resulted in remarkable ideas, such as immunosensors for Chagas’ disease antibodies and detecting lymphocytes and the total content of immunoglobulin [135,137]. 

An IgG–IgM immunochromatographic assay was developed for the detection of IgG and IgM against SARS-CoV-2 while using antihuman antibodies as biorecognition components. This simultaneous and rapid detection method was able to give a response within 15 min with sensitivity and specificity of 85.29% and 100.00% [138]. Piotr et al., demonstrated the detection of goat IgG by using biotin–avidin–biotin interactions and antibody anti-goat IgG interactions [139]. Wang et al., studied the importance of the Mach–Zehnder interferometer (MZI) for the detection of IgG. A low detection limit interferometric optical fiber biosensor for IgG/anti-IgG immunosensing was proposed [47]. 

#### 2.3.2. Aptamer-Based IgG Detection

Genosensors, also known as DNA/RNA sensors, are small analytical instruments that employ genetic material analysis to identify pathogens, such as bacteria and viruses, illnesses, and/or propensity to diseases [140]. Immobilized DNA/RNA probes are used as a recognition element in nanosensors, allowing particular hybridization reactions to occur, often via DNA–DNA or DNA–RNA molecular recognition [140]. Biosensors are not limited to antibodies as biorecognition elements but may also utilize other elements such as DNA or RNA (Aptamers) with the same transduction capabilities [136]. Nucleic acids, deoxyribonucleic acid (DNA), and ribonucleic acid (RNA) convey genetic information that is read in cells to produce RNA and proteins for normal human body function [141]. 

Aptamers are single-stranded oligonucleotides that fold into certain structures and bind to specific targets, such as proteins. They frequently block protein–protein interactions in binding proteins, which may result in therapeutic effects, such as antagonism [141]. As extremely specific strands have been observed against a wide range of biomarkers, nucleic acid aptamers offer significant potential as diagnostic reagents. They provide enticing advantages, such as repeatable creation by chemical synthesis, regulated modification with labels and functions that provide varied ways for detection and directed immobilization, and strong biochemical and thermal resistance. Aptamers against immunoglobulin targets (IgA, IgM, IgG, and IgE) have a distinct diagnostic domain; hence, multiple aptamers have been identified and employed in conjunction with diverse detection modalities [142]. According to another study, immunosensors use DNA and RNA as biorecognition elements by simultaneously detecting two tumor markers, carcinoembryonic antigen (CEA) and alpha-fetoprotein (AFP) [137]. It is also useful in the identification of autoimmune disorders with IgG by detecting early molecular processes that were not discovered by previous techniques, such as physician assessment [143]. 

#### 2.3.3. Enzyme-Based IgG Detection

Enzymes are biological catalysts (also known as biocatalysts) that help living organisms accelerate metabolic processes [144]. Enzymes are catalysts composed of a lengthy chain of amino acids linked together by a peptide bond, commonly known as protein. These are utilized to start any reaction by adhering to a specified spot on the substrate. Enzymes are specific in their function, and their activity is affected by substrate concentration as well as other physical factors, such as temperature and pH [145]

Enzymes are an important biorecognition material for biosensors [146]. Enzyme-based biosensors measure alterations in proton concentration (H+), gas release or uptake (e.g., CO_2_, NH_3_, O_2_, etc.), light emission, absorption or reflectance, heat emission, and other factors that happen during substrate consumption or product formation of enzymatic reactions to detect the presence of analytes. The transducer then converts these variations into quantifiable signals (electrical, optical, or thermal signals) that can be used to identify potentially interesting analytes [147]. However, there are many techniques for using enzymes in biosensors: the enzyme converting the analyte into a sensor-detectable product; detection of an enzyme blocked or activated via the analyte; and monitoring enzyme property alteration [148]. Recent research demonstrates how enzyme-based biosensors have been successfully applied in IgG detection. For example, given the quick catalytic reaction and redox cycling, the detection limit for mouse IgG and prostate-specific antigen was reduced to 1 fg/mL [149]. According to the literature, using hydrazine, EC redox cycling was applied in an ALP-based biosensor to reduce p-quinone imine [129]. Finally, the pandemic increased the inclusion of enzymatic biosensors in the industry as SARS-CoV-2 uses enzyme-linked immunosorbent assay (ELISA) to detect IgG levels in the blood to determine the type of SARS-CoV-2 [150]. 

#### 2.3.4. Biomimetic IgG Detection

Biomimicry is an applied science that draws inspiration for human issue solutions from the study of natural designs, processes, and systems [151,152]. Fluid-drag reduction swimsuits inspired by the structure of shark skin, airplane shapes inspired by the appearance of birds, low-drag wind turbines inspired by humpback whales, and sturdy building constructions inspired by the backbone of turban shells are some examples of biomimetic investigations [153]. This method of seeking inspiration from nature for human applications is also applied in biosensors.

Biomimetic biosensors use artificial receptors, also known as biocompatible materials, that are not sourced from biological creatures but have compositions and qualities that are comparable to those made by living species [154]. They are utilized as sensors in tissue reactions, bone cement in bone-bonding systems, joint replacements, skin restoration devices, and drug delivery, to highlight a few applications [154]. Successful biomaterials either: (1) restore a natural function where the original material is missing or unable to perform as intended or (2) maintain an environment that is most suited for procedures such as cell culture, tissue growth, biomolecular assays, and biotechnology-based manufacturing [155]. An example of biomimetic biosensors is the use of heterogeneous catalysis of imine hydrogenation [156]. A similar device was used to quantify a carcinoembryonic antigen (CEA) and -fetoprotein using antibodies that had been marked with gold nanoparticles (AFP) [157]. A highly sensitive glycoprotein sensor based on molecularly imprinted polymers (MIP) that were electropolymerized in the presence of template molecules (bovine serum albumin) is another application (BSA) [158]. 

Dianping et al., proposed a biomimetic interface based on glucose oxidase for the detection of IgG. The biomimetic interface was fabricated onto a gold electrode by its modification with polydendrime, Au/SiO_2_ nanocomposite, Fc-anti-IgG antibodies, and GOx (glucose oxidase) as a backfiller. Electrochemical testing as well as human sample testing were performed to check the reproducibility, sensitivity, and selectivity of an IgG sensor with a reported detection range from 3.5 × 10^−5^ to 1.2 × 10^−3^ mol/L [130]. These applications of biorecognition elements show immense potential in improving the biomedical industry and improving the cost and accessibility of diagnostics in the future via interdisciplinary biorecognition element utility.

## 3. Conclusions and Future Perspective

The presented review emphasizes the significance of various biosensors for the detection of IgG antibodies and their working principles, as IgG plays a crucial role in different pathological conditions and its use as a biomarker tool for diagnostic applications. Here, a comparison has been made between the use of biosensors with respect to the transduction properties in mass-based biosensors (magnetoelectric, piezoelectric, quartz-crystal microbalance, and surface acoustic waves) and biorecognition elements (antibodies, aptamers, enzymes, and biomimetic materials). The initial discussion was based on the classification of biosensors while highlighting the advantages and drawbacks of electrochemical biosensors (potentiostatic, conductometric, impedimetric, and amperometric) with an in-depth literature review of how biosensors have evolved over the decade. Biosensors, especially electrochemical biosensors, have seen major developments over the years with their initial use as glucose sensors for IgG detection in SARS-CoV-2. Although the sensors utilize similar principles, they differ in sensitivity, response rate, cost, and biochemical and thermal resistance using diverse biomaterials or a pair of biorecognition materials with a complimentary transducer. Biosensors offer applications to multiple industries for drug delivery, food safety monitoring, toxicology, medicine, and technology; however, with IgG biosensors, we have seen a popular demand in disease prevention for diagnosis of prostate cancer, tumor markers, autoimmune diseases, and COVID-19. Although IgG biosensors have many advantages, they do have certain disadvantages, such as detection limit, detection time, selectivity, and difficulty in maintaining a high throughput system. Many of these issues are controllable thanks to recent advances in nanomaterial production, such as carbon nanotubes, nanodiamonds, and metallic organic frameworks. Furthermore, the balance between specificity and versatility is problematic because single IgG molecules are used by an expensive biosensor to maintain specificity, and to make affordable biosensors, IgG detection with protein molecules must be expanded using adaptable machine learning models that are programmed iteratively, leading to a more efficient method of IgG detection. Finally, with more suitable nanomaterials and well-designed software, IgG detection could be possible in handheld devices that can be utilized for point-of-care detection in every home.

## Figures and Tables

**Table 1:** Comparison of various kinds of biosensors based on transduction mechanisms for IgG detection.

Biosensor Type	Biorecognition Element	Transduction Platform and Material Used	Application Area	Advantages	Detection Range (LOD)	Ref.
Electrochemical biosensor	Y-shaped peptides	PEDOT-citrate/AuNPs modified electrode	Antifouling biosensor for the detection of IgG	Remarkable performance with high specificity, sensitivity, stability, and selectivity	100 pg mL^−1^ to 10 μg mL^−1^ (32 pg/mL)	[40]
	Protein G	Microgap parallel plate electrode	Highly reproducible biosensing	Higher reproducibility than conventional interdigitated electrode sensing	1 × 10^–13^ to 1 × 10^–7^ mol/L (f 1 × 10^–14^ mol/L)	[41]
	Measles-specific antigen	Gold surface	Serodiagnosis of measles	Good stability of coated antigens	1 μg mL^−1^	[42]
	Label free	ZnO@ZIF-8/IL composite film	Composite film for the detection of nitrite and IgG	Highly selective and good reproducibility	0.1–10 and 10–400 ng/mL (0.03 ng/mL)	[43]
		Rigid biocomposites	Biosensor for rabbit IgG detection	First antigen–antibody ratio-based assay	---	[44]
Optical biosensor	Goat antihuman IgG	HD microdisk (Active WGM)	Optical sensor for practical and research applications	Incredibly low detection limit and a benchmark for high throughput systems	0.007 [a_M_]–0.667 [m_M_] (0.06 [a_M_])	[45]
	Label-free binding assay	High index contrast polymer material system	Suitable alternative to inorganic optical biosensors	Cost effectivity	5–200 nM (3.1 nM)	[46]
	Secondary antihIgG antibody (H + L)				Down to 100 pM (30 pM)	
	Staphylococcal protein A and goat antihuman IgG	Single-mode fiber with large core-offset fusion splice based on Mach–Zehnder interferometer	Sensor for biomedical applications	Low detection limit with simple fabrication method and higher sensitivity. Simpler fabrication, high sensitivity, low detection limit, reusability.	(47 ng/mL)	[47]
	Label free	A correction system based on a nonimprinted polymer (NIP)-coated LPFG	Highly selective and specific transduction platform for the detection of biomolecules	Reliability and good sensitivity	0.25 nmol/L 1 to 100 nmol/L,	[48]
	Label free	GO-SPA-modified TFBG-SPR biosensor	Biosensor for biochemistry field	Small-size, label-free, and sensitive biosensor	0.096 dB/(μg/mL) and 0.5 μg/mL (0.5 μg/mL)	[49]
	Protein A and goat antihuman IgG antibody	Graphene oxide/silver film SPR	Sensor for future biomedical and biochemistry applications	High sensitivity with real-time monitoring low detection limit with multiple usabilities	0.4985 nm/(μg/mL) (0.04 μg/mL)	[50]
	Goat antihuman immunoglobulin G (IgG)	Two different sensing channels with different modifications	---	No temperature sensitivity, high accuracy, and sensitivity	15 ng/mL	[51]
	Goat antihuman IgG	Au film-coated photonic crystal fiber (Au-PCF) with gold NPs and protein A	---	Good recognition performance and sensitivity	37 ng/mL	[52]
	Alkyne-terminated peptides	Thermoresponsive hydrogel attached to the metallic surface through SAM of linker molecule	Signal enhancement in fluorescence biosensor	Higher fluorescence up to five folds	---	[53]
	Protein AG-Cys	Meta surface sensor system	Dual channel fiber biosensor	High throughput with good sensitivity	5 pg/mL	[54]
Solution-gate FET biosensor	Ovalbumin molecules	Silicon nanowire-based	Early detection of diseases	Rapid and ultrasensitive detection	6 aM to 600 nM (6 aM)	[55]
Mass-based biosensor	Protein A	125 MHz AT-cut quartz resonators	Biosensor for potential application in clinical disease diagnosis	Cost effective, low power requirement, good reliability, and sensitivity.	1 ng mL^–1^ or less	[56]

**Table 2:** Comparison of various kinds of biosensors based on biorecognition elements for IgG detection.

Transduction Platform and Material Used	Detection Mechanism	Advantages	Detection Range (LOD)	Ref.
Antibody and proteins-based IgG biosensors				
Fab-modified CNT–FET	Field emission transistor (FET) based detection	Low LOD as compared to biosensors using whole antibodies	(∼7 fM level)	[121]
Lanthanide-doped polystyrene nanoparticles (LNPs)	Lateral flow immunoassay	Identification of suspicious cases, good for monitoring and evaluating progress of diseased condition upon treatment	---	[122]
Multiplexed grating-coupled fluorescent plasmonic platform	Fluorescent immunoassay	Sensitivity and selectivity up to 100%	---	[123]
Aptamer-based IgG biosensors				
Silicon nanowire	FET based assay	Immunoassay for both direct and sandwich detection of rabbit IgG	---	[124]
DNA assisted nanopores	LFA	Reliable quantification, high accuracy, automated assay with dynamic range	---	[125]
Aptamers were designed to study their specificity	SPR analysis	Good selectivity of rabbit IgG	---	[126]
MXene with bovine serum albumin (BSA)	Electrochemical detection	Quantitative as well as sensitive detection	0.1 ng/mL to 10 µg/mL (23 pg/mL)	[127]
Enzyme-based IgG biosensors				
Enzyme paper-based biosensor	Amperometry and chemiluminescence (CL)-based detection	Suitable for point-of-care detection	(12 fM)	[128]
One-electrode, one-enzyme format	Electrochemical Immunosensing	Significantly low limit of detection	100 fg/mL to 100 μg/mL	[129]
Biomimetic IgG biosensors				
Glucose oxidase-based biomimetic interface	Flow injection electrochemical detection	Acceptable sensitivity, selectivity, and reproducibility.	3.5 × 10^−5^ to 1.2 × 10^−3^ mol/L (--)	[130]

## Data Availability

Not applicable.
